# Charge transport mechanisms and memory effects in amorphous TaN_*x*_ thin films

**DOI:** 10.1186/1556-276X-8-432

**Published:** 2013-10-17

**Authors:** Nikolaos Spyropoulos-Antonakakis, Evangelia Sarantopoulou, Goran Drazic, Zoe Kollia, Dimitrios Christofilos, Gerasimos Kourouklis, Dimitrios Palles, Alkiviadis Constantinos Cefalas

**Affiliations:** 1 National Hellenic Research Foundation, Theoretical and Physical Chemistry Institute, 48 Vassileos Constantinou Avenue, Athens 11635, Greece; 2 Laboratory for materials electrochemistry, National Institute of Chemistry, Hajdrihova 19, Ljubljana 1000, Slovenia; 3 Physics Division, School of Technology, Aristotle University of Thessaloniki, Thessaloniki 54124, Greece

**Keywords:** Nitrides, TaN_*x*_ thin films, Amorphous semiconductors, Nanoelectronics, Memory effects, Conductive-AFM

## Abstract

Amorphous semiconducting materials have unique electrical properties that may be beneficial in nanoelectronics, such as low leakage current, charge memory effects, and hysteresis functionality. However, electrical characteristics between different or neighboring regions in the same amorphous nanostructure may differ greatly. In this work, the bulk and surface local charge carrier transport properties of a-TaN_*x*_ amorphous thin films deposited in two different substrates are investigated by conductive atomic force microscopy. The nitride films are grown either on Au (100) or Si [100] substrates by pulsed laser deposition at 157 nm in nitrogen environment. For the a-TaN_*x*_ films deposited on Au, it is found that they display a negligible leakage current until a high bias voltage is reached. On the contrary, a much lower threshold voltage for the leakage current and a lower total resistance is observed for the a-TaN_*x*_ film deposited on the Si substrate. Furthermore, I-V characteristics of the a-TaN_*x*_ film deposited on Au show significant hysteresis effects for both polarities of bias voltage, while for the film deposited on Si hysteresis, effects appear only for positive bias voltage, suggesting that with the usage of the appropriate substrate, the a-TaN_*x*_ nanodomains may have potential use as charge memory devices.

## Background

Nowadays, there is an urgent need of efficient dielectrics with minimum leakage current as electronic devices are shrinking to only few nanometers. A class of nanomaterials that display these characteristics is amorphous semiconductors [1]. Generally, amorphous semiconducting nanostructures display some advantageous electrical characteristics compared with their crystalline counterparts. In particular, due to their disordered structure, amorphous materials typically have a high density of localized defect states, resulting in significant charge trapping and much lower leakage current [2]. Moreover, amorphous nanomaterials can be produced at relatively low temperatures, while a lower strain is expected between the embedded nanoparticles and the matrix due to their flexible amorphous structure [3]. In addition, very recent works have demonstrated that some amorphous or polycrystalline nitrides, like CuN, AlN, and NiN, exhibit resistive switching behavior capable for fabricating resistance-switching random access memory devices [4-7]. However, the research for switching resistive materials had been focused almost only on metal oxides, e.g., TiO_2_[8,9], NiO [10,11], ZnO [12], and Ta_2_O_5_[13-16], as their electrical properties are well known and their preparation methods are relatively easy and well established. On the contrary, metal nitrides, even though they exhibit intriguing electrical properties, remain largely unexplored in this field.

Low-power memristive behavior with outstanding endurance has been already demonstrated in tantalum oxide [13-15], alongside with efforts to maximize its performance with nitrogen doping [16]. A promising material in this point of view is amorphous tantalum nitride (a-TaN_*x*_). Tantalum nitride is proved to be a mechanically hard and a chemically inert material, combining both high thermal stability and low temperature coefficient of resistance [17,18]. TaN_*x*_ appears with many crystalline phases that are well studied [19,20]. For example, the metallic TaN may have potential applications as Cu diffusion barriers [21], thin film resistors [22], and superconducting single-photon detectors [23], while nitrogen-rich Ta_3_N_5_ is used as photocatalytic material for water splitting [24,25]. On the other hand, the amorphous phase (a-TaN_*x*_), which is the most common phase of the as-prepared TaN_*x*_ at relatively low temperatures [26-28], has received very low attention. Early electrical studies on a-TaN_*x*_ films by Chang et al. showed that there was increasing resistivity of films, as the nitrogen concentration in the gas environment increased [29], while Kim et al. [30] indicated that a-TaN_*x*_ could prevent copper diffusion more effectively than the crystallized Ta_2_N film by eliminating grain boundaries.

It is well known for 1-D and 2-D nanostructures, i.e., nanowires, nanorods, and thin films, that the electrical properties may differ greatly from point to point within regions separated by several nanometers, due to differences in charge concentration, defect density, surface band bending, etc. [31,32]. It is also established that the large surface-to-volume ratio of these nanostructures results in increasing contribution of the surface and space-charge-limited current to the total current [33]. Hence, local measurements with the conductive atomic force microscopy (C-AFM) technique are of high importance, because C-AFM is capable of resolving the electrical properties at the nanoscale.

In this letter, the local charge carrier transport mechanisms and memory effects of a-TaN_*x*_ thin films deposited either on Au (100) or Si [100] substrates by pulsed laser deposition (PLD) at 157 nm [34] are investigated by C-AFM, and the influence of the space charge layer in conductivity along with a pronounced current hysteresis is revealed. For the sample’s characterization, atomic force microscopy (AFM), focused ion beam (FIB), transmission electron microscopy (TEM), micro-Raman spectroscopy, and energy-dispersive X-ray spectroscopy (EDXS) are used.

## Methods

a-TaN_*x*_ films are prepared by PLD at 157 nm (LPF 200, Lambda-Physik, (since 2006 Coherent, Santa Clara, CA, USA)) in a vacuum stainless steel chamber at ambient temperature under 10^5^ Pa of research grade (99.999%) N_2_ gas. The pulsed discharged molecular fluorine laser at 157 nm has been used previously in various applications where high energy per photon is required [34-36]. A high-purity tantalum foil (99.9%, Good-Fellow, Huntingdon, UK) of 0.5 mm in thickness is used as the ablation target. The films are efficiently deposited using relative low laser energy per pulse (30 mJ) with 15-Hz repetition rate. The pulse duration is 15 ns at full width at half maximum. The Au (100) or Si [100] substrate is placed approximately 3 to 5 mm away from the target material and perpendicular to the optical axis of the laser beam in axial ablation geometry. In previous works, PLD at 157 nm has been used to grow metal nitrides efficiently [37-39].

An AFM (d’Innova, Bruker, Madison, WI, USA) is operated at ambient conditions to evaluate the morphology and roughness of the as-deposited a-TaN_*x*_ films. The AFM images are acquired in tapping-mode using a phosphorus-(n)-doped silicon cantilever (RTESPA, Bruker, Madison, WI, USA) with a nominal spring constant of 40 N/m at approximately 300-kHz resonance frequency and nominal radius of 8 nm. The AFM images are obtained at different scanning areas at a maximum scanning rate of 0.5 Hz with an image resolution of 512 × 512 pixels. FIB technique with a Pt protection layer is used to determine the film thickness, while TEM (operated at 200 kV; Jeol 2100, JEOL Ltd., Akishima-shi, Japan) is carried out to reveal the different structures in TaN_*x*_ deposited on Si. In order to be examined in the microscope, the samples are transferred to a lacey-carbon-coated Cu grid. Additionally, EDXS with a Si(Li) detector from JEOL is performed to detect the nitrogen and oxygen content, while micro-Raman spectroscopy using the 488-nm line of an Ar^+^ laser, which irradiates a sample area of 1 μm^2^ with a power of 3 mW, is performed at room temperature to identify the possible crystalline or amorphous phases.

**Figure 1:**
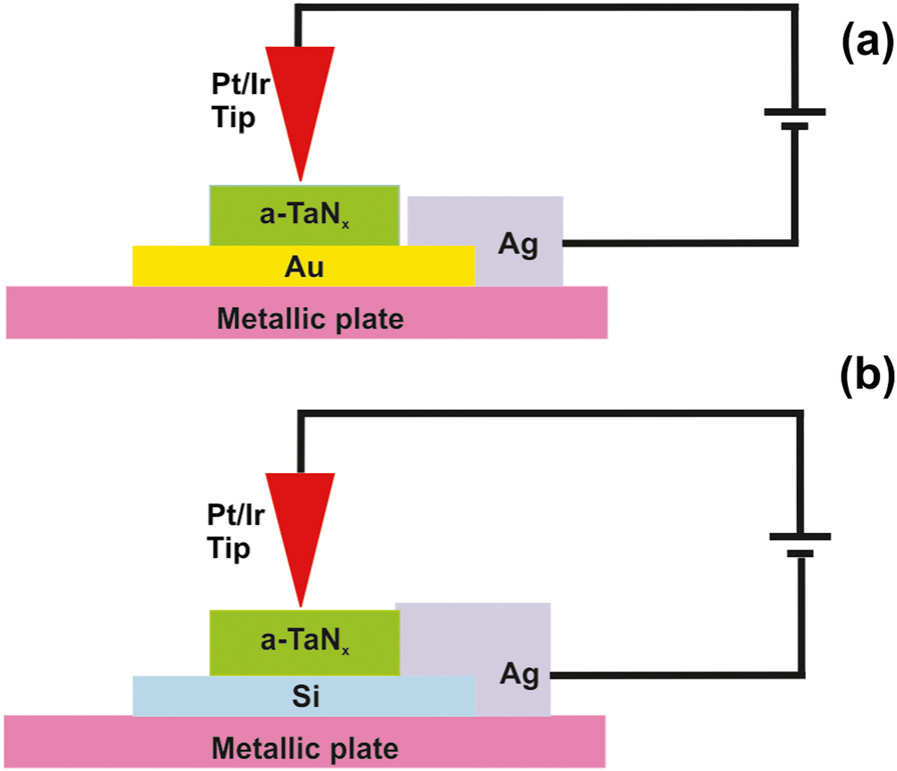
**Simplified diagrams of C**-**AFM and devices. (a)** The Pt/Ir-TaN_*x*_-Au device. **(b)** The Pt/Ir-TaN_*x*_-Ag device.

The charge transport properties of the a-TaN_*x*_ nanodomains are evaluated with a C-AFM (d’Innova, Bruker). A Pt/Ir-coated tip (SCM-PIC) of conical shape with tip radius approximately 8 nm, spring constant 0.2 N/m, and resonant frequency 13 kHz is used as the top metal electrode, resulting in a 10-nm^2^ effective contact area. A strip of conductive silver paint bridges the metal–semiconductor-metal junction with the AFM circuit when the substrate is the metallic Au, and it plays also the role of the bottom electrode in the case of the Si substrate. The simplified circuits of Pt/a-TaN_*x*_/Au and Pt/a-TaN_*x*_/Ag devices are illustrated in Figure 1a,b, respectively. The tip is kept on virtual ground, while a pre-selected bias voltage is applied between the tip and the sample to avoid anionic oxidation. A femto-gain amplifier, with a gain factor of 10^7^ in the case of TaN_*x*_ deposited on Au and 10^8^ in the case of TaN_*x*_ deposited on Si, is used to detect the low C-AFM signal.

## Results and discussion

**Figure 2:**
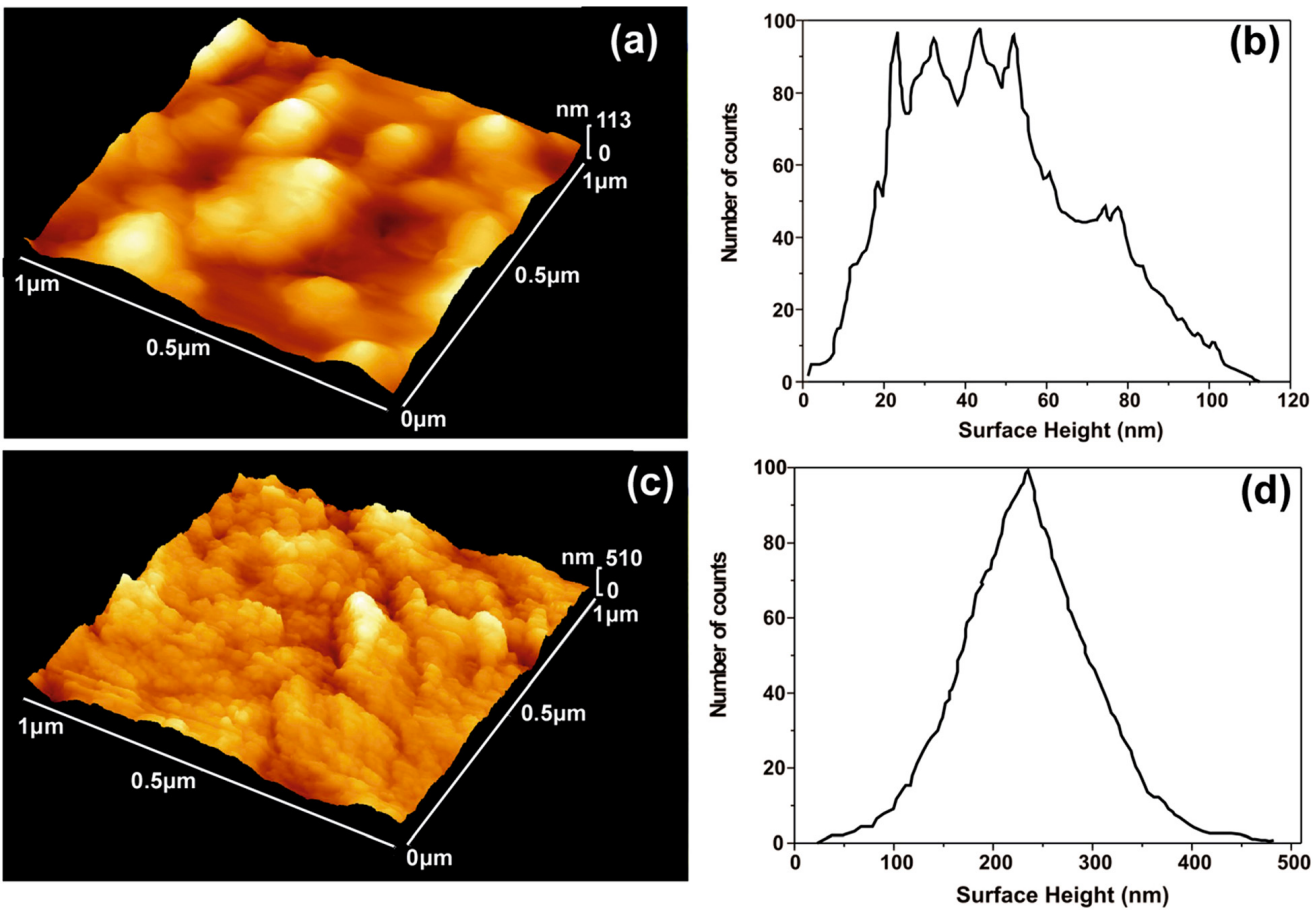
**Surface morphology of TaN**_***x***_**with AFM imaging. (a)** AFM mapping of the TaN_*x*_ film on Au substrate reveals smooth round-shaped nanoislands. **(b)** The corresponding histogram shows that the average roughness is 48 nm. **(c)** AFM mapping of the TaN_*x*_ film on Si substrate reveals grainy nanoislands with high roughness consisting of smaller nanoparticles. **(d)** The distribution of the film’s roughness is shown with average of 248 nm.

Different morphological features of the a-TaN_*x*_ films deposited on Au and Si are displayed by the AFM topological mapping. For the a-TaN_*x*_ deposited on Au, the film consists of relative smooth round-shaped nanoislands with average surface roughness of 48 nm and root of middle square (RMS) of 22 nm, as it is shown in Figure 2a,b. Whereas, for the a-TaN_*x*_ deposited on Si, the film consists of larger nanoislands with average surface roughness of 248 nm and RMS of 68 nm, which are created by the agglomeration of smaller grains, as it is shown in Figure 2c,d. Because the deposition parameters of both films are the same except for the type of the substrate, the above results indicate that a-TaN_*x*_ agglomeration is affected by the substrate [39].

**Figure 3:**
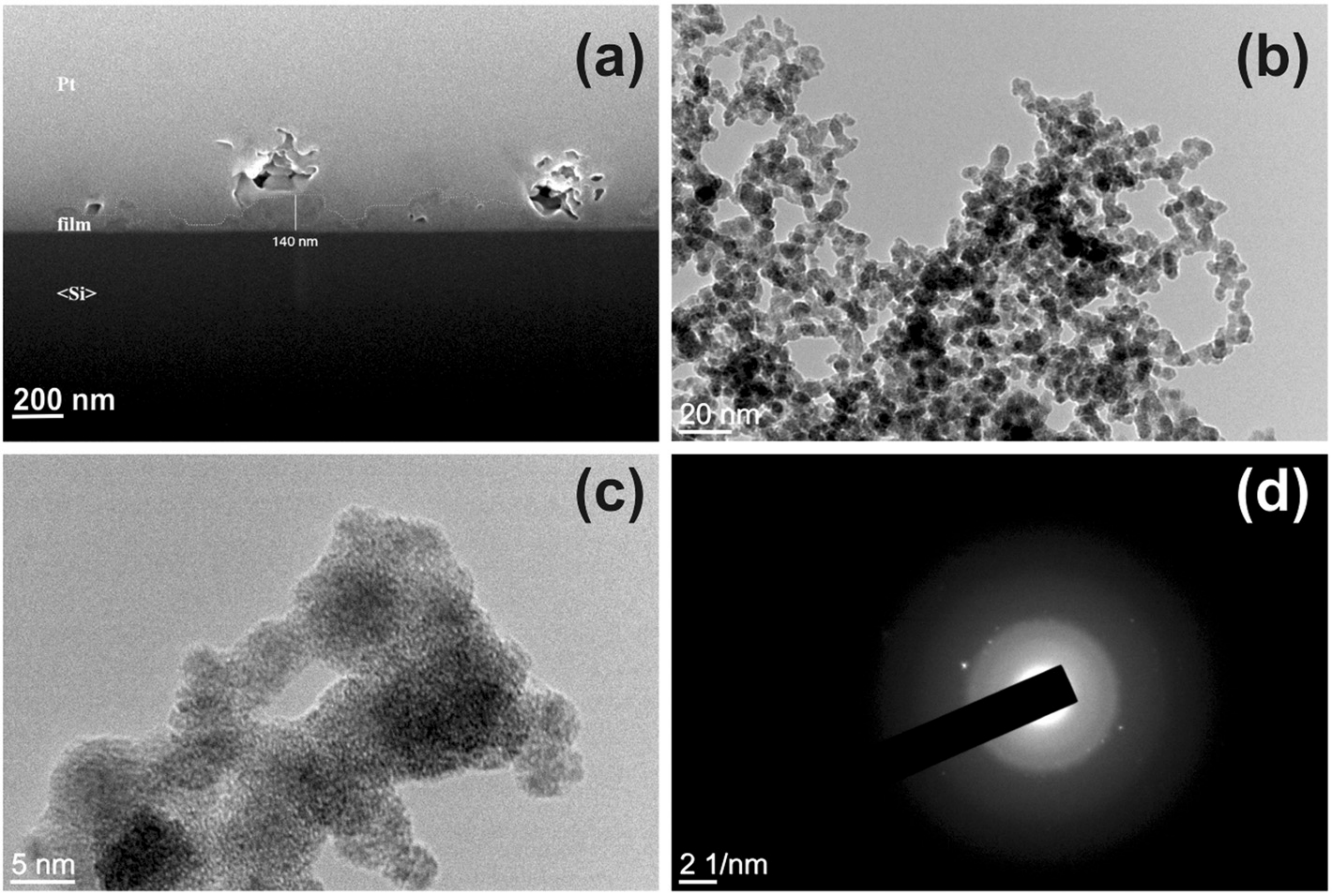
**FIB and TEM images of the TaN**_***x***_**film deposited on Si. (a)** Cross section of the TaN_*x*_ film deposited on Si obtained with FIB technique. **(b)** TEM image of amorphous and chain-like structures. **(c)** HRTEM image of 5-nm nanoparticles forming the chain-like structure. **(d)** Selected-area electron diffraction (SAED) pattern, where beside the diffused broad band characteristic for amorphous material, faint spots are present which could be indexed as cubic Fm-3m tantalum.

**Figure 4:**
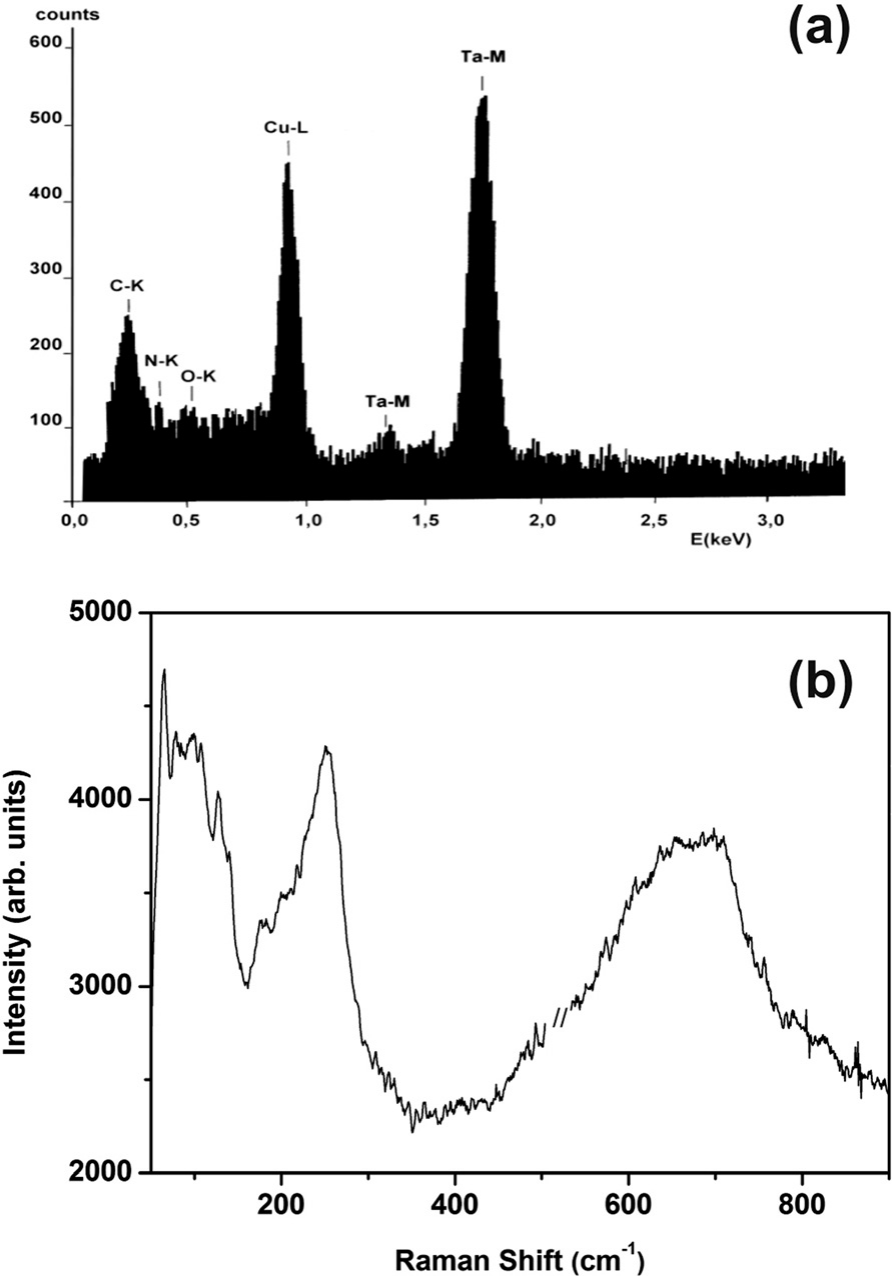
**EDXS and micro**-**Raman spectrum of TaN**_***x***_**deposited on Si. (a)** EDXS spectrum. The presence of nitrogen verifies the formation of a-TaN, and the concentration of oxygen is lower than the detection limit (few wt. %). **(b)** Raman spectrum of TaN_*x*_ on Si. The broad peaks indicate the amorphous character of the film.

In Figure 3a, a typical FIB cross section of the TaN_*x*_ thin film deposited on Si is shown. The darkest layer above the Si substrate corresponds to the TaN_*x*_ layer with maximum thickness of the film to be around 140 nm. Amorphous, chain-like nanostructures in the TaN_*x*_ film deposited on Si are identified by TEM, Figure 3b, and they are composed from the agglomeration of individual nanoparticles with 5-nm mean diameter, as the high-resolution transmission electron microscopy (HRTEM) image of Figure 3c illustrates. The selected-area electron diffraction pattern of Figure 3d reveals the presence of few metallic Ta nanocrystals embedded in the otherwise amorphous semiconducting matrix. EDXS analysis of the samples evidently reveals nitrogen and tantalum peaks, verifying the formation of tantalum nitride, Figure 4a. Meanwhile, the concentration of oxygen is lower than the detection limit (few wt.%), excluding the unintentional formation of tantalum oxide or oxynitride phases, Figure 4a. Furthermore, in Figure 4b, the broad bands of the Raman spectra from 60 to 140 cm^-1^ and from 590 to 720 cm^-1^ suggest that TaN_*x*_ film is formed on Si substrate and it is amorphous in nature, while the Raman shift around 250 cm^-1^, not reported in the literature for the TaN_*x*_ films with *x* < 1.37 [40,41], indicates that a N-rich phase might be present. For the films deposited on Au, it was impossible to detect Raman spectra due to the strong luminescence from the Au substrate. However in this case, the amorphous phase is confirmed visually as the samples have the characteristic distinctive yellow-brown color of the amorphous tantalum nitride [42]. The correlation between color and composition in TaN_*x*_ is well known, as highly conductive tantalum nitrides (*x* ≤ 1) have been reported to be gray, whereas semiconducting crystalline Ta_3_N_5_ (*x* ≈ 1.66) is red and semiconducting amorphous TaN_*x*_ is yellow-brown [28].

**Figure 5:**
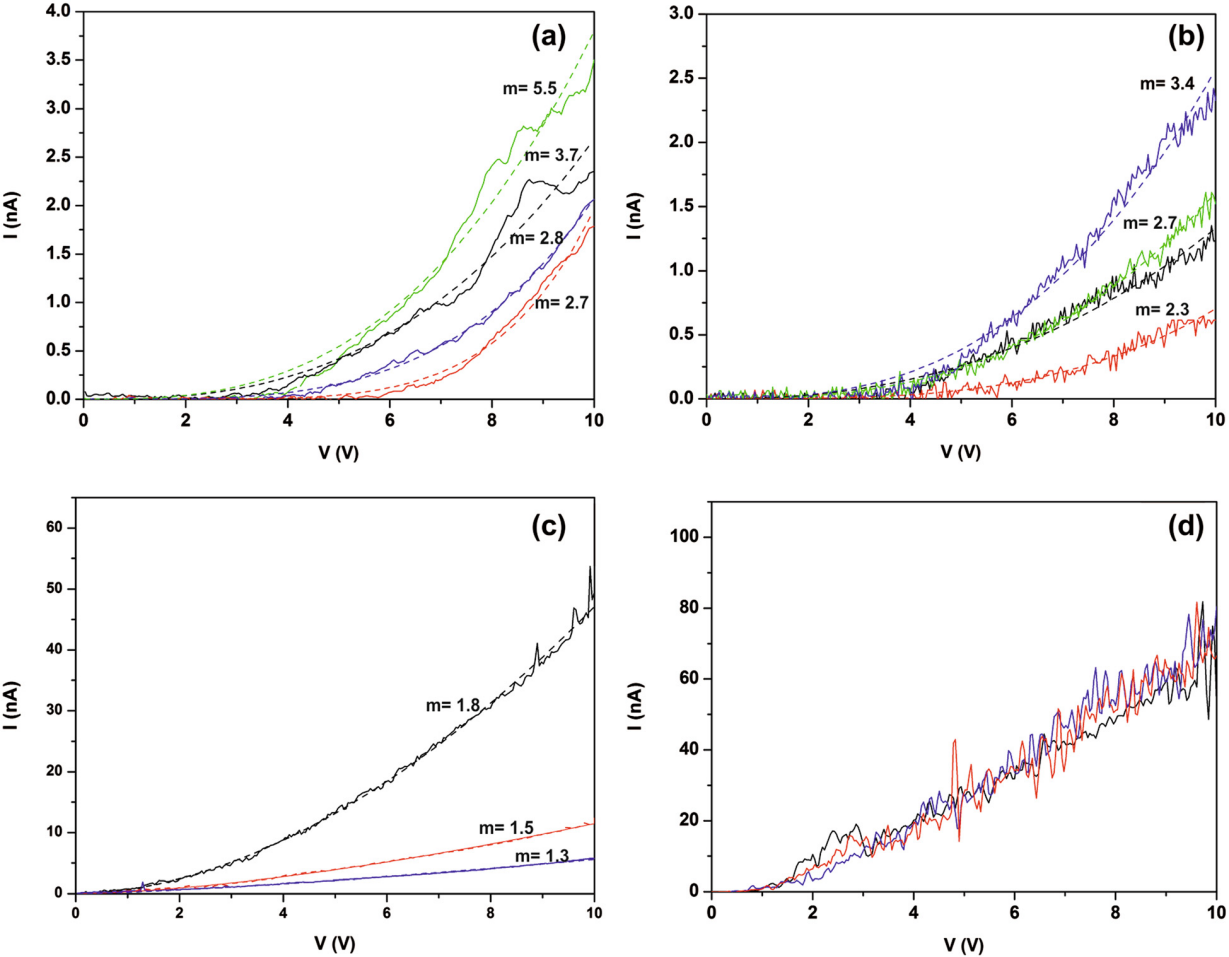
**C-AFM measurements of a-TaN**_***x***_**. (a)** Positive I-V curves (solid lines) of TaN_*x*_ deposited on Au for four different points fitted by the space-charge-limited current (SCLC) model (dash lines). **(b)** Negative I-V curves (solid lines) of TaN_*x*_ deposited on Au for the same points presented in **(a)** fitted by the SCLC model (dash lines). **(c)** Positive I-V curves of TaN_*x*_ deposited on Si for three different points. The conductive part of the I-Vs exhibits an almost parabolic to almost ohmic behavior **(d)** Negative I-V curves of TaN_*x*_ deposited on Si for the points presented in **(b)**. In all I-Vs, the leakage current is quite high, displaying also a very noisy profile.

By fixing the tip on individual nanodomains of a-TaN_*x*_ films deposited on Au or Si, the local I-V characteristics are repeatedly recorded with the voltage being swept from -10 to 10 V. In Figure 5, the I-V curves for forward and reverse bias voltages at several local points are shown for TaN_*x*_ deposited on Au (Figure 5a,b) and Si (Figure 5c,d). At first glance, comparing the I-Vs of the nanodomains, which are located on the same film, small or large differences in conductivity and threshold voltage are observed for both films. However, the shape of the I-Vs is quite similar, indicating that the conduction mechanism is the same for all nanodomains located on the same film. Comparing now, the I-Vs between the two films, it is seen that the threshold voltage for the leakage current at the point contacts of Figure 5a is higher than 3.5 V, while for the point contacts in Figure 5c, the threshold voltage does not exceed 1 V. It is also noticed that there is a different response of the I-Vs in the two metal-dielectric-metal devices.

In general, the total current flowing through a semiconductor can be written as *I*_tot_ = *I*_b_ + *I*_s_, where *I*_b_ is the current from the bulk part of the film and *I*_s_ includes the electronic conduction through the surface states and through the space charge layer beneath the surface. Taking into account the amorphous nature of the semiconducting film, the main conduction mechanism from the bulk is expected to be the Poole-Frenkel effect [43]. Usually in amorphous materials, the predominant conduction mechanism is the Poole-Frenkel effect, i.e., the thermal emission of electrons from charged vacancies, attributed to impurities and defects that are present in large numbers inside the bulk of the amorphous matrix [43,44]. In the present samples, charged nitrogen vacancies act like Coulombic traps that promote the injection of electrons from the Au or Ag bottom electrode as the electric field increases during forward bias direction and from Pt/Ir tip during the reverse bias direction. For Poole-Frenkel emission, the current density is given by [45]:

(1) $$J=\mathrm{CE}\text{exp}\left[-\left(q\varphi -\frac{\beta \sqrt{E}}{\text{kT}}\right)\right]$$

where *C* and *β* are material dependent constants, *E* is the induced electric field, *q* is the electron charge, *T* is the temperature, *k* is the Boltzmann constant, and *φ* is the ionization potential in *V*. The constant *C* is related to charge carrier mobility and trap’s density, while *β* is related to the dielectric constant *ε*_0_*ε*_*r*_ via

(2) $$\beta =\sqrt{\frac{q^{3}}{\pi \epsilon_{0}\epsilon_{r}}}$$

Other possible charge carrier transport mechanisms from the bulk of the film could be thermionic emission of charge carriers across the metal-dielectric interface or field emission by electron tunneling from the metal or charge traps to the quasi-conduction band of the amorphous semiconductor [46]. These mechanisms have also exponential like I-V behavior.

However, after the examination of the I-V curves in Figure 5, it is found that they exhibit a power-like (Figure 5a) to almost linear (Figure 5c) response rather than the characteristic exponential behavior of the above charge carrier transport mechanisms, indicating that the current contribution from the bulk is small compared to the space charge and surface current. The I-Vs in Figure 5a are fitted well by a power law *I* ∝ *V*^*m*^, with *m* = 2.7 to 5.5, indicating that the predominant charge carrier transport mechanism is the space-charge-limited current [47-50]. Due to the band bending of the quasi-conduction band near the metal-dielectric interfaces, a space charge layer is formed near the surface of the dielectric where electrons are depleted. Hence, under a voltage threshold, the electrons injected from the gold electrode are combined with the holes which are present in the space charge layer resulting in the decrease of free carriers. With the increase of voltage bias, the holes are fully filled after a voltage threshold, causing the rapid increase of free carriers. Similar results are obtained for the I-V characteristics under negative bias, where *m* = 2.3 to 3.4, Figure 5b.

On the contrary, the a-TaN_*x*_ film deposited on Si, despite it is thicker than the film deposited in Au, displays much lower voltage threshold, lower total resistance, and parabolic to almost linear current behavior for higher bias voltages, Figure 5c. This is attributed to the presence of tantalum nanoparticles, as those identified in Figure 3d, which provide additional free charge carriers after a proper value of the applied field, changing the conductive behavior from almost parabolic, *m* = 1.8, to almost ohmic, *m* = 1.3 to 1.5, Figure 5c [49,50]. The threshold value of the applied field is much lower compared to the a-TaN_*x*_ deposited on Au, considering the lower threshold bias voltage and the thickness of the film. Furthermore, all the I-V characteristics under negative bias show a quite high leakage current with a very noisy profile, although the mean current still has a linear dependence to the voltage bias (Figure 5d). This high flow of electrons under negative voltage bias may be attributed to the usage of a low work function bottom electrode (Ag, *φ* = 4.5 eV) compared with the high work function electrode (Au, *φ* = 5.1 eV) that is used in the other device. The charge transport at the metal-dielectric interface depends on the Schottky barrier height (SBH) which is defined as *φ*_b_ = *φ*_m_ - *χ*, where *φ*_m_ and *χ* are the metal work function and electron affinity of the dielectric, respectively. Hence, in the case of an n-doped dielectric, lower metal work function results in lower SBH and easier charge transport through the barrier.

**Figure 6:**
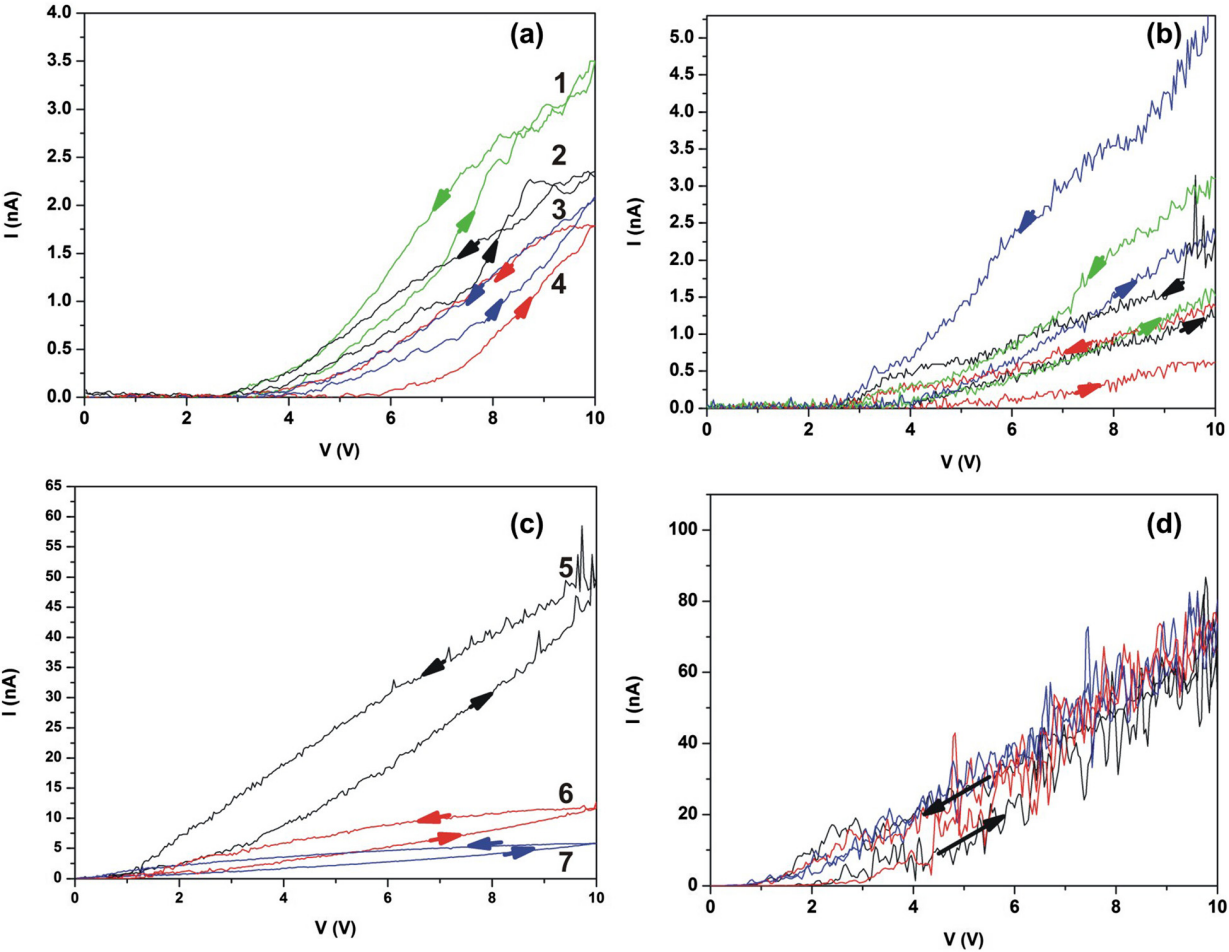
**Double sweeping of voltage bias on different nanodomains of both a**-**TaN**_***x ***_**films. (a)** Positive and **(b)** negative voltage bias swept on four nanodomains (curves 1 to 4) of a-TaN_*x*_ film deposited on Au. **(c)** Positive and **(d)** negative voltage bias swept on three nanodomains (curves 5 to 7) of a-TaN_*x*_ film deposited on Si. In the first three figures, significant current hysteresis is observed, while in the last figure, hysteresis effects are negligible.

Next, the two devices are double swept from -10 to 10 V to detect possible hysteresis phenomena, Figure 6. Indeed, pronounced current hysteresis of the retrace during the forward and reverse biasing cycle of the tip is identified only for the a-TaN_*x*_ film on Au. The hysteretic loops are attributed to the conservation, during the bias voltage decrement process, of the internal electric fields caused by the stored space charges near the surface. Hysteresis, in this work, is defined as delta I at a fixed voltage. In Table 1, the hysteresis for the several I-V loops along with the calculated resistivity ratio at 3.5-V bias voltage where the resistivity ratio is quite high is shown. In Figure 6a,b, it is shown that all measured local points for a-TaN_*x*_ deposited on Au, despite they demonstrate different conductivity, exhibit significant current hysteresis for positive and negative bias voltage. In contrast, for a-TaN_*x*_ deposited on Si, positive voltage sweeping results in a resistivity ratio smaller than 3, Figure 6c, while hysteresis of the I-Vs for negative voltage sweeping is negligible, Figure 6d. This is consistent with the observed high-current and the low-voltage threshold, previously mentioned, which indicate low charge storage in that film.

**Table 1:** **Hysteresis and the calculated resistivity ratio at 3**.**5**-**V bias voltage**

**Point contact (Figure**6**, curves 1 to 7)**	**Hysteresis [ *δI *** **(nA)] at 3.5 V**	**Resistivity ratio at 3.5 V**
1 a-TaN_*x*_ on Au	0.4	>80
2 a-TaN_*x*_ on Au	0.2	>40
3 a-TaN_*x*_ on Au	0.2	>40
4 a-TaN_*x*_ on Au	0.5	>100
5 a-TaN_*x*_ on Si	9.4	2.5
6 a-TaN_*x*_ on Si	2.7	2.2
7 a-TaN_*x*_ on Si	1.8	2.3

## Conclusions

In summary, it is found that the conduction on metal/a-TaN_*x*_/metal devices through the amorphous film is dominated by the space-charge-limited current and the current contribution from the bulk is small compared to the space charge and surface current. The conduction of the devices is also expected to be greatly influenced by the eventual presence of Ta nanoparticles embedded in the amorphous matrix and the choice of the metal electrodes, as it is shown in the case of the a-TaN_*x*_ films deposited on Si. Large variations between neighboring nanodomains on the same film are found. These variations in conductivity between nanodomains of the same film establish the importance of C-AFM technique as a diagnostic tool in nanoelectronics. Finally, significant current hysteresis effects are demonstrated, indicating the possible use of a-TaN_*x*_ in memory applications, especially for a-TaN_*x*_ deposited on Au where bipolar memory effects are observed.

## Competing interests

The authors declare that they have no competing interests.

## **Authors**’ **contributions**

NSA participated in the design of the study, helped with C-AFM, interpreted the results, analyzed the micro-Raman spectra, and wrote the manuscript. ES conceived and designed the study, performed C-AFM, and participated in micro-Raman spectroscopy. GD carried out the TEM imaging and analysis. ZK participated in C-AFM. DC, GK, and DP performed micro-Raman spectroscopy. ACC conceived the work and participated in the study. All authors read and approved the final manuscript.
